# New Appliances and Things Medical

**Published:** 1894-08-04

**Authors:** 


### NEW APPLIANCES AND THINGS MEDICAL.

[All preparations, appliances, novelties, &c., of which a notice is desired, should be sent for the Editor, to care of The Manager, 428, Strand, London, W.C.]

### SANITARY FLUID AND SANITARY POWDER.

(Whalley and Co., Limited, 56 and 57, Aldermanbury, London, E.C.)

Both of the above-mentioned antiseptic media have secured considerable popularity, a popularity which is certainly well deserved, since they both contain a high degree of efficacy, with an absence of danger unusual to most of the powerful antiseptics known to the public and the profession. The sanitary fluid, which may be obtained in bottles of about half a pint, or in casks containing 40 gallons, is a thick tarry substance which readily mixes with water, forming a milky solution, and has the characteristic smell of a coal distillate. When thus diluted it forms a valuable antiseptic for scrubbing down floors, washing walls, or cleansing sinks and traps, the fresh, clear odour of this fluid rapidly replacing any previously existing disagreeable smell. The anti septic powder, which is sold in tins with perforated lids, forming a sort of giant flour dredger, is eminently suited for use in public lavatories and domestic water-closets. Its systematic and daily use in the latter will satisfy all sanitary requirements as far as can be secured by antiseptic methods. Both the fluid and the powder have extensive veterinary application, and will be found valuable adjuncts both to the stable and farmyard.

